# RF Characterization of GaAs HBT under Load Mismatch with Reverse Wave Injection Technique

**DOI:** 10.3390/mi14112058

**Published:** 2023-11-03

**Authors:** Yidong Xu, Yuxiu Tong, Jiangtao Su

**Affiliations:** 1 Qingdao Jari Industrial Control Technology Co., Ltd., Qingdao 266000, China; 18205813884@163.com; 2 Key Laboratory of RF Circuits and Systems, Ministry of Education, Hangzhou 310018, China

**Keywords:** RF PA, HBT, load mismatch, VSWR

## Abstract

RF PAs need to be reliable enough to protect them from damage under load mismatch conditions. This paper investigated the characteristics of GaAs heterojunction bipolar transistors (HBTs) under load mismatch conditions using a novel reverse wave injection technique to realize large VSWR ruggedness measurement with the circle centered at 50 Ohm and optimal impedance separately to analyze the device in real applications. With a real-time waveform measurement system, the RF voltage and current waveform information can be acquired, which provide a more-accurate view of what is occurring at the current generator plane of the HBT device. Thereby, the potential failure mechanisms and load impedance can be identified to design the most-suitable PA circuits in communication systems.

## 1. Introduction

GaAs-based heterojunction bipolar transistors (HBTs) have long been recognized as the leading device technology for high-speed high-power applications. They have also gained extensive usage in modern commercial and military communications equipment, mainly serving as high-efficiency amplifiers in portable communication devices. Their exceptional attributes, such as high linearity, high current gain, and low noise, contribute to their popularity [1]. While HBT-based RF circuits are normally designed to operate on a nominal load equal to the system impedance, in various wireless environments, the antenna’s operational conditions are uncontrollable, leading to the impedance mismatch with a voltage standing wave ratio (VSWR) greater than 1. This impedance mismatch significantly reduces the device’s output power and efficiency and further decreases the performance of communication systems. Furthermore, as in all bipolar devices, HBTs suffer from various feedback phenomena, which may cause instability and device failure in certain operating conditions, such as self-heating [2,3,4].

To address impedance mismatch issues, conventional practice involves placing an isolator between the power amplifier (PA) and the antenna. The isolator safeguards the transistor from high current and voltage fluctuations during load mismatch, ultimately improving device performance [5,6]. Despite its effectiveness, this approach proves impractical for handheld devices due to the isolator’s bulkiness, cost, and adverse impact on transmission power. Therefore, it becomes imperative to comprehensively understand the mechanism of device breakdown under strong load mismatch and identify a safe operational region for the target application [7].

A widely adopted method for assessing a device’s RF robustness is testing the device under constant-voltage standing wave ratio (VSWR) conditions [8]. It is a critical test for PA circuit development as mismatch is often the main cause of failure for PAs. With the help of this test, protection mechanisms or device technology can be designed to tolerate high-VSWR conditions. More recently, the VSWR sweeping technique has been evolving to improve the sweep area and its ability to diagnose failures when they occur. That is to say, the data obtained from a VSWR sweep help diagnose what caused the device to fail during the sweep, which can then be used to make the technology more robust or give information to the PA designers to make sure impedances that may cause the device to fail will not be seen by the device.

The traditional load mismatch test technology is based on a passive impedance tuner. However, this technology typically can only cope with a 5:1 VSWR circle mismatch test [9]. Even if the loss between the impedance tuner and the device under test (DUT) is reduced to the minimum, the impedance reflection coefficient is still limited to 0.8 or less. Recently, the real-time active load pull technique has been proposed to replace passive-tuner-based systems, which is able to test a VSWR mismatch as high as to 10:1 [10]. However, today, most of the VSWR test circles are typically centered at 50 ohms, which represents the standard impedance. Hence, this may not accurately mirror real-world scenarios, where devices typically operate under optimal load impedance [11]. To rectify this, an alternative approach has been proposed using the VSWR circle with the optimal impedance point as the center for testing load impedance mismatch performance [12]. Therefore, the ability to move the impedance away from the optimum as much as possible is important to simulate the effects of stressing the HBT device in real applications.

In this paper, a real-time waveform-measurement system with the reverse wave injection technique is used to perform VSWR testing of a GaAs HBT device. It shows how the addition of reverse wave injection can increase the maximum VSWR circles the system can obtain and the acquired RF IV waveform, allowing the exact state of the device to be known under RF operation, which is helpful for the identification of potential failure locations. Moreover, to the best of the authors’ knowledge, this is the first time that the VSWR circles have been actually tested on non-50 Ohm center using the active wave injection technique. This modified test method aligns more closely with practical applications and offers valuable insights into the device’s performance under realistic impedance conditions.

## 2. Mismatch Issues of HBT

### 2.1. HBT Theory

Semiconductor devices can be categorized into bipolar transistors (HBTs/BJTs) and field-effect transistors (FETs/HEMTs). In a bipolar transistor, both electrons and holes play a role in carrier transport, and the device’s amplification capability is determined by the current amplification factor. For N-type transistors, the B–E junction is positively biased during the normal operation of a BJT [9]. Due to the high impurity concentration in the emitter, a large number of electrons diffuse from the emitter into the base, while a small number of free electrons recombine with holes, most of which become non-equilibrium minority carriers in the base and reach the collector.

**Figure 1:**
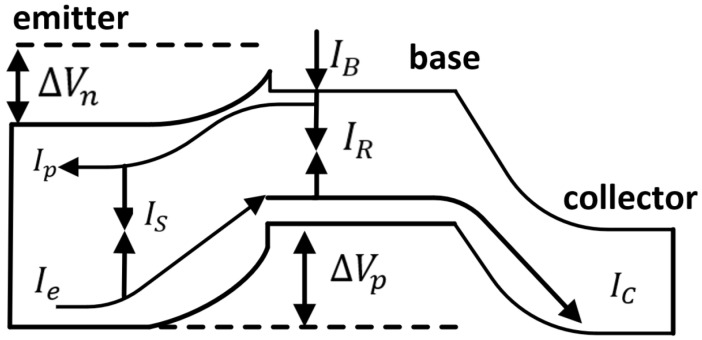
HBT energy band and internal current schematic.

The most-significant difference between HBTs and BJTs lies in the introduction of a wide bandgap emitter in HBTs. The base and collector are made of narrow bandgap materials, while a wide bandgap material is used in the emitter region. This creates a heterojunction between the B–E interfaces [13]. In HBTs, the wide bandgap emitter material reduces the barrier for electrons injected from the emitter into the base compared to the barrier for holes injected from the base into the emitter at the emitter–base interface. This improvement in electron injection enhances the forward current of the base, reduces the reverse current, significantly enhances the current gain, and promotes higher emitter efficiency. Furthermore, the inherent difference in conduction bandgaps eases the trade-off between gain and frequency performance [14]. Figure 1 depicts the energy band diagram and internal current flow schematic of HBTs, where $I_{B}$ and $I_{C}$ represent the base and collector currents, respectively, and $I_{p}$ and $I_{e}$ represent the hole current injected from the base region into the emitter region and the electron current injected from the emitter region into the base region, respectively. $I_{R}$ and $I_{S}$ represent the bulk recombination current in the base region and the recombination current in the space charge region of the heterojunction, respectively.

### 2.2. HBT Breakdown Characteristics

For HBT devices, it is noted that the base–collector junction will have a larger voltage differential across it than the emitter–base junction. The voltage across the base–collector is also reverse biased; both of them cause the junction fail due to voltage stress. Furthermore, within the junction, these high reverse bias voltages would cause impact ionization in the depletion region, which potentially will cause an avalanche effect.

Avalanche breakdown can further cause lattice damage due to the impact ionizations or cause thermal damage due to the increased currents flowing. For the common emitter configuration, which is how the devices used in this paper were configured, the breakdown voltage will be reduced compared to common base mode, and therefore, peak RF voltage failure is a particular concern for HBTs in common emitter mode.

Another cause of breakdown within HBT devices is thermal run-away, which is where an increased junction temperature leads to the bandgap energy on the materials decreasing, and so, the base–emitter junction potential drops. This would, in turn, cause the emitter current to increase. When the device is operating at high voltages, the current would bend over on itself, and if a device has multiple fingers, then some fingers may go into the high-current state and others into the low-current state. As a result, the high-current fingers will heat up and are likely to thermally run away.

Both avalanche and thermal run-away can feed into each other and can be triggered by high voltages on the output of the device. Therefore, when the load of the HBT is mismatched, a large voltage is generated at the collector of the device, which may cause damage to the HBT. Because of that, the transistor voltage withstanding test is an important issue to consider in the design of the PA. Furthermore, when performing reliability tests on the HBT device, it is important to be able to observe the RF current and voltage waveforms to understand the possible breaking mechanism. For example, if the impedance of the device bias circuit is high, i.e., the base is driven by the current, the collector–base breakdown current is fed back to the base–emitter junction, thereby increasing the voltage at the base–emitter. The collector current increases, and thus, breakdown occurs due to thermal run-away. Otherwise, if the bias circuit driving the RF transistor has a low impedance, i.e., a voltage source is applied to the base, the collector–base breakdown current is initially shunted by the external base bias resistor and breakdown occurs at a voltage higher than that of the $V_{BVCEO}$ (the collector to base breakdown voltage).

### 2.3. VSWR Test

The concept of power waves was introduced to deal with both complex load and source impedances in real applications [15]. The degree of matching (or rather mismatch) can be measured by the reflection coefficient for power waves, i.e., $Gamma_{L}$, as

(1) $$\Gamma_{L}=\frac{z_{L}-z_{0}^{*}}{z_{L}+z_{0}}$$

where $z_{L}$ is a load impedance and $z_{0}$ is the reference impedance. When dealing with traveling waves, mismatch can be expressed in different ways, such as Gamma, return loss (reflection coefficient expressed in decibels), and the VSWR. The voltage standing wave ratio (VSWR) is defined as the ratio of the amplitude of the standing wave antinode voltage to the trough voltage amplitude. A VSWR value of 1 indicates a perfect impedance match between the device and the load. Under this condition, all the energy is efficiently transmitted without any loss due to reflected energy. As the VSWR value increases, it signifies a more-severe impedance mismatch between the device and the load. At the extreme, when the VSWR becomes infinite, this indicates total reflection, where no energy is transmitted at all [16]. The relationship between the VSWR and reflection coefficient can be expressed by (2). As can be seen, the PWR is any possible transformation of the power wave reflection coefficient to a parameter ranging from 1 to infinity.

(2) $$VSWR=\frac{1+\Gamma_{L}}{1-\Gamma_{L}}$$

When evaluating the RF robustness of a device, the test is usually performed at a constant VSWR circle centered at 50 ohm. However, the optimum impedance point of a device is usually a complex impedance, and in practical applications, the device is usually matched at the optimal load impedance point. Therefore, it is more interesting to evaluate the robustness around the optimal load impedance, which is more in line with the actual working environment of the device [17]. In this case, we can determine the trace of the VSWR circle using Equations (3) and (4), where $\Gamma_{c}$ is the center of the circle and *R* is the radius.

(3) $$\Gamma_{c}=\Gamma_{opt}\frac{1-cte^{2}}{1-cte^{2}{\left|\Gamma_{opt}\right|}^{2}}$$

(4) $$R=cte\frac{1-|\Gamma_{opt}{|}^{2}}{1-cte^{2}{\left|\Gamma_{opt}\right|}^{2}}$$

where cte is $cte=\frac{VSWR-1}{VSWR+1}$. $\Gamma_{opt}$ represents the reflection coefficient of the optimal impedance point. As can be seen, with this method, the sweep area in Smith chart is often larger than the constant VSWR circle centered at 50 ohm. For design and technology purposes, the VSWR ruggedness investigation has to, therefore, be performed at the transistor level with as large a reflection coefficient as possible.

### 2.4. Proposed Reverse Wave Injection Method

Conventionally, the VSWR test is achieved using basic passive load pull systems. The signal $b_{2}$ generated by the device is inserted into the passive tuner, where the phase shifter and the variable attenuator alter its phase and magnitude, respectively. The modified signal $a_{2}$ is then inserted back to the device output. This setting of the reflection coefficient determines the impedance $Z_{load}$ seen by the device to be

(5) $$\Gamma_{L}=a_{2}/b_{2}$$

However, despite the simplicity of the passive load pull, its application for the VSWR test is rather prohibitive. The tuner has to be disconnected at each setting, and its reflection coefficient needs to be pre-calibrated by a vector network analyzer (VNA). Considering that the test of a device requires several tens if not hundreds of points at several bias conditions and frequencies, it becomes obviously very time consuming.

Another weakness of passive-tuner-based systems is that they only provide scalar performance parameters such as output power, gain, and efficiency. The actual voltage and/or current values are not known, and therefore, if a device does fail, there is no RF I–V information to help identify the source of the failure. It is, therefore, necessary to develop more-advanced system that allow the RF I–V waveform information to be measured to help identify the cause of the failures.

**Figure 2:**
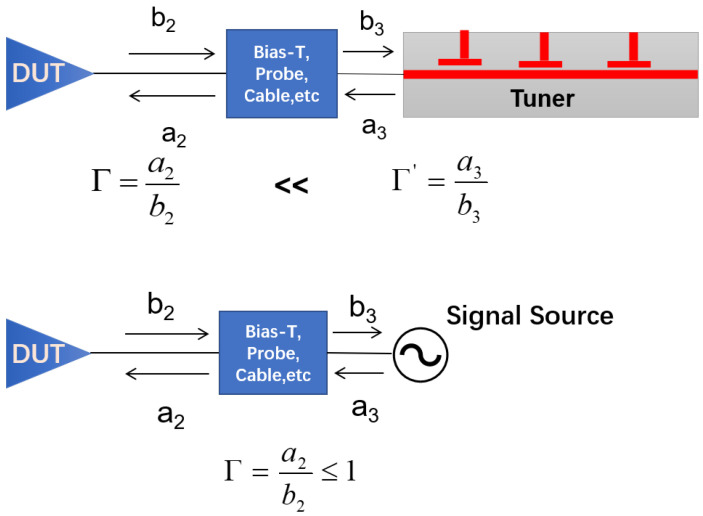
The difference between the tuner-based system and the proposed reverse wave injection system.

In this paper, we propose the reverse-wave-injection-based method as a substitution for passive tuners. The mechanism of this technique is illustrated in Figure 2. As can be seen, for tuner-based systems, the accumulated components such as the bias-tees, probes, and cables inserted between the passive tuners and the device inevitably reduce the maximum magnitude of the modified reverse signal $a_{2}$ and, thus, limit the reflection coefficient that can be achieved at the device output. Actually, the desired load reflection coefficient can be achieved by actively injecting the signal at the DUT output port. The value of the injected signal, $a_{3}$, is solely responsible for the load synthesis, and hence, a load reflection coefficient of even higher than unity can also be achieved using reverse wave injection; thereby, this technique helps in overcoming the limitations imposed by the passive load pull system.

## 3. Measurement Setup

**Figure 3:**
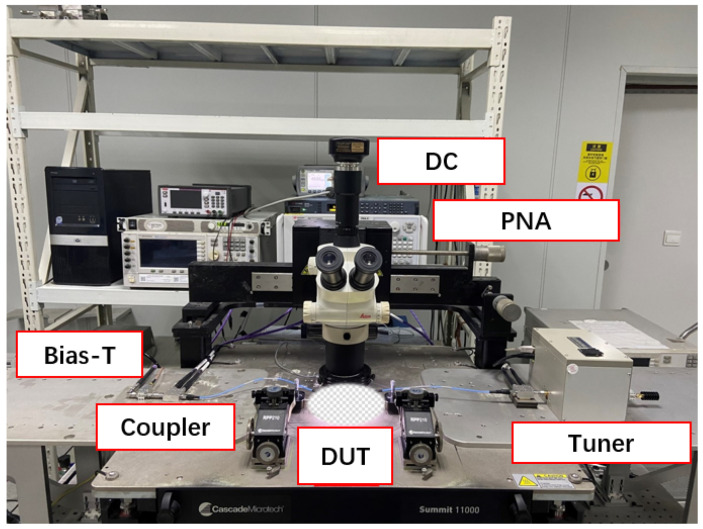
Tuner-based VSWR test system.

To realize the VSWR load mismatch test, we first employed a traditional tuner-based test system, as illustrated in Figure 3. The tuner is strategically placed between the device under test (DUT) and the bias-tee to minimize loop loss, thereby maximizing the achievable impedance range. This system is renowned for its high-power-handling capacity, cost-effectiveness, and excellent system stability. However, the limited range of impedance that the tuner can synthesize is a primary drawback of tuner-based systems. Moreover, the presence of the tuner prevents real-time data acquisition, resulting in limited information when it comes to understanding the root cause of device breakdowns.

**Figure 4:**
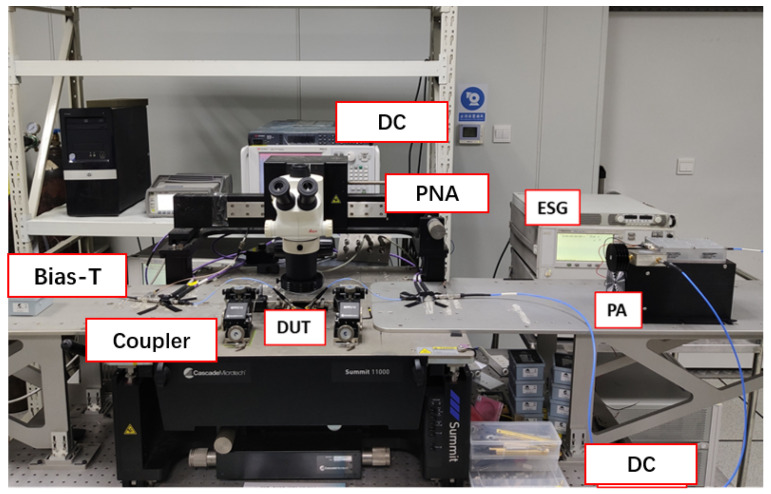
Reverse-wave-injection-based VSWR test system.

**Figure 5:**
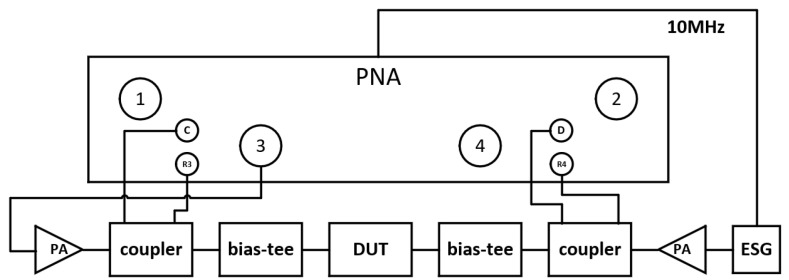
Simplified calibration topology diagram. The number 1 to 4 represents the ports of VNA.

A real-time reverse-wave-injection-based system for VSWR testing, as introduced in Section 2, is depicted in Figure 4 and Figure 5 for the actual test system, and a system block diagram, respectively, is proposed to tackle the issues of tuner-based systems. As can be seen in the figure, we utilized an electronic signal generator (ESG) and power amplifier (PA) to create an active impedance tuner [18]. The output signal of the ESG is amplified by the PA to form the incident wave at the load port of the device. By manipulating the signal’s amplitude and phase, we can synthesize the required reflection coefficient, accurately simulating the load impedance. It can be seen that the signal to synthesize at the load side is referenced to the input signal with a 10 MHz local signal from the VNA; therefore, the realized load impedance is stable over time. The synthesized impedance range of this system surpasses that of traditional tuner-based systems. Also, attaching the source to the device output creates no active loop; loop oscillations were eliminated, which makes it suitable for the application of the VSWR test due the high power that needs to be injected to achieve unity impedance.

Additionally, the test system employs external bi-directional couplers to measure the incident and reflected waves, which are fed into the VNA to provide shorter test paths for the incident and reflected waves compared to the conventional approach using the internal couplers of the VNA. As a result, the loss of incident and reflected waves in the transmission path is minimized, leading to more-precise and -stable wave testing by the receiver. Moreover, we can enhance the test system’s upper power limit by incorporating an attenuator on the coupler, thus relying on the upper limit of the RF probe test power. The MPI T50 Ground-Signal-Ground (GSG) RF probe was used in this experiment, which has a power limit of 5 W (approximately 36.99 dBm), effectively improving the test power upper limit of the system. To apply DC bias to the DUT, two BT-0040 Bias-Tees from Marki Microwave, are connected to the DC source (Keysight B1500A), manufactured by Keysight Technologies in Santa Rosa, CA, USA.

**Figure 6:**
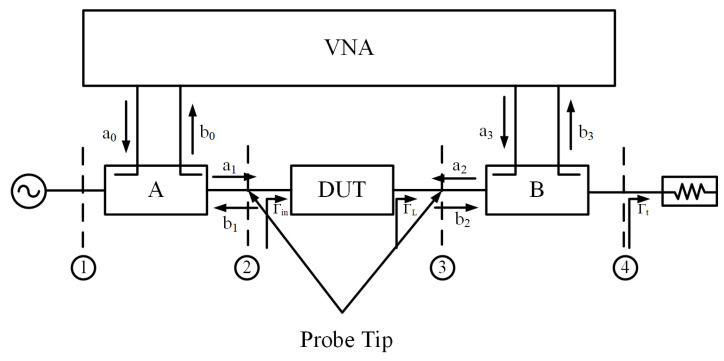
Block diagram of proposed reverse wave injection measurement system. The number 1 to 4 represents four measurement planes.

To calibrate the large signal measurement system shown above, a simple topology diagram is drawn in Figure 6, where $a_{0},b_{0},a_{3},b_{3}$ are the measured traveling waves and $a_{1},b_{1},a_{2},b_{2}$ are the actual traveling waves into and out of the DUT. Block A and Block B are the error boxes representing the errors from the two ports of the DUT to the VNA receivers. Obviously, accurate measurements of these traveling waves at the DUT ports require calibration, which will be explained in the following sections, to remove the errors introduced by the dispersion and losses in the system components, fixture, and test set defined by the error boxes.

**Figure 7:**
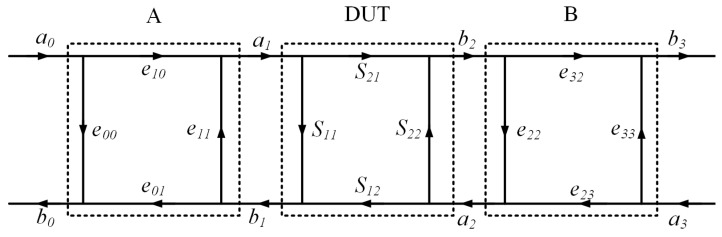
Generic measurement calibration model.

The above diagram can be further abstracted to a generic error model, as shown in Figure 7, where $e_{00},e_{01},e_{10},e_{11}$ are the error terms of Block A and $e_{22},e_{23},e_{32},e_{33}$ are error terms of Block B. We can use an 8-term error model [16], as shown in Equation (6), to describe the calibration process to determine the 8 error terms from the measured $a_{0},b_{0},a_{1}$, and $b_{1}$ waves, which consist of a set of measured uncorrected s-parameters over the calibration standards, as well as power measurement results.

(6) $$\left(\begin{array}{c} b_{1}\\ a_{1}\\ b_{2}\\ a_{2} \end{array}\right)=\frac{1}{e_{01}}\cdot \left(\begin{array}{cccc} b_{1} & -e_{00} & 0 & 0\\ e_{11} & \left(e_{01}e_{10}-e_{11}e_{00}\right) & 0 & 0\\ 0 & 0 & \frac{1}{F_{32,01}} & -\frac{e_{33}}{F_{32,01}}\\ 0 & 0 & \frac{e_{22}}{F_{32,01}} & \frac{e_{32}e_{23}-e_{33}e_{22}}{F_{32,01}} \end{array}\right)\cdot \left(\begin{array}{c} b_{0}\\ a_{0}\\ b_{3}\\ a_{3} \end{array}\right)$$

where $F_{32,01}=\frac{e_{32}}{e_{01}}=\frac{e_{32}e_{01}}{e_{01}e_{10}}$.

The calibration process consists of small-signal calibration and power calibration. For small-signal calibration, we adopted the thru–reflect–match (TRM) method [19]. This calibration technique enabled us to characterize the transmission line accurately and correct any systematic errors introduced by the test setup. Subsequently, we measured various data points, including the input wave, reflected wave, and current–voltage (I–V) curve of the DUT. By analyzing these test data, we can derive key parameters such as the output power, gain, and efficiency of the DUT, thereby comprehensively evaluating its performance under varying load conditions.

The VNA measures the Fourier coefficients A(w) and B(w) of a two-port device simultaneously, but one frequency at a time. These measurements, which occurred at different harmonics, may be agnostic of anaffixed time trigger. This leads to the relative phase of the waveforms’ frequency being unknown. Hence, the simple addition of a synchronization component is necessary to generate the correct time domain waveform. This aim was achieved by using a common time reference during the swept frequency VNA measurements. More specifically, the reconstruction of the signal in the time domain, therefore, required some means of measuring, directly or indirectly, the phase of the LO, again relative to a common time reference. Using an HPR generator in the measurement system (the VNA with the HPR generator to construct the VNA) gave us the ability to measure the system to reconstruct the signal in the time domain. In this paper, a comb generator was used as the HPR generator with a similar time domain waveform reconstruction method as introduced in [20].

## 4. Measurement Results

**Figure 8:**
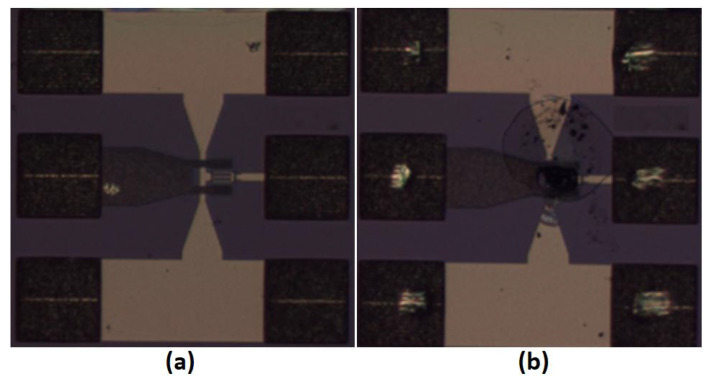
Picture of the HBT device under test: (**a**) 2 × 2 × 20 HBT device, (**b**) Broken device.

In this paper, an HBT device with a size of 2 × 2 × 20 was selected for the VSWR testing. The device is shown in Figure 8a. We set the bias condition as $I_{b}=0.15$ mA and $V_{C}=5$ V for the initial optimum output power impedance and power sweep test. Then, $V_{C}$ was gradually increased from 5 V until the breakdown of the device while undergoing the VSWR test. The appearance of the device after the breakdown is shown in Figure 8b.

The whole measurement process consisted of the following steps: Firstly, the pinch-off test was conducted, which was performed by sweeping the gate voltage (VG) from the pinch-off until a maximum drain current. This test was used to identify the temporary or permanent pinch-off voltage (VP) shift from VSWR stress. After that, output characteristic (I–V) was measured for a limited number of gate voltages to identify a change in the maximum drain current (IDMAX) and possible punch-through.

After the above initial static DC tests, a power sweep, where the load impedance was fixed at 50 Ohm, is conducted first to find the large-signal working status, e.g., the transistor is compressed. The load pull test was then carried out to find the optimum impedance. Taking the optimum impedance as the center, the constant VSWR impedance circles were calculated, and the reverse wave injection technique introduced in this paper was used to move the impedance to the locus of the VSWR circles.

**Figure 9:**
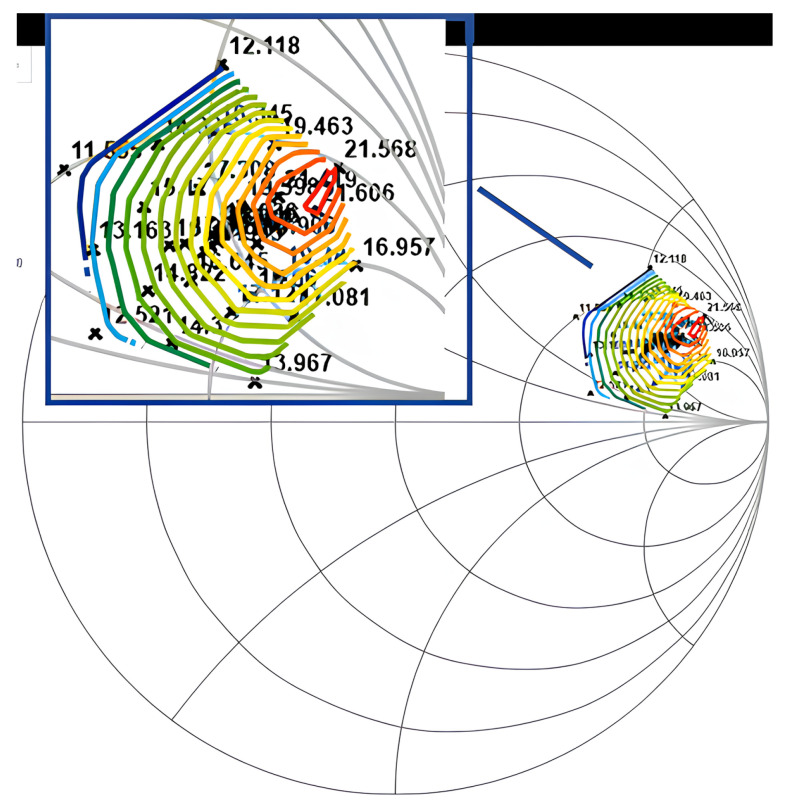
Output power contours of the HBT device (dBm).

**Figure 10:**
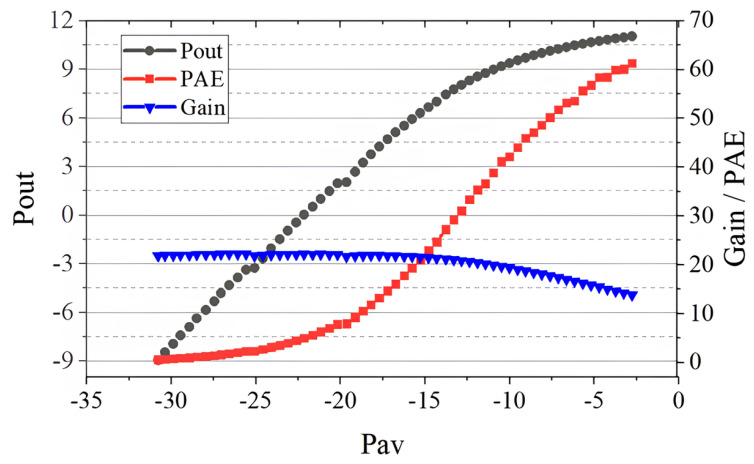
Performance of the device at impedance matching.

Firstly, we performed the power sweep to find the P1dB compression point when the load impedance was 50 Ohm. Then, we performed a load pull test at this input power to find the optimal impedance. The test results are shown in Figure 9, where the output power contours show that the load impedance when the Pout was maximum was (165.7+j×252.1) Ohm. We can obtain the performance of the device at impedance matching by performing the power sweep test at this point, and the test results are shown in Figure 10. At the P1db compression point, the transducer gain was 21.07 dB, the output power was 7.77 dBm, and the power added efficiency (PAE) was 29.23%.

**Figure 11:**
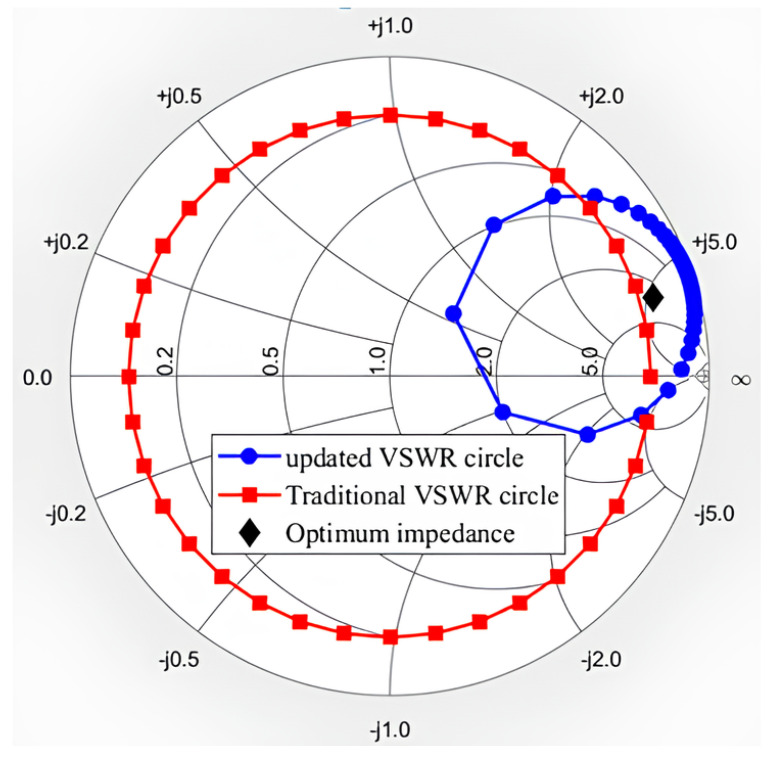
Impedance points for VSWR = 10 under different conditions.

**Figure 12:**
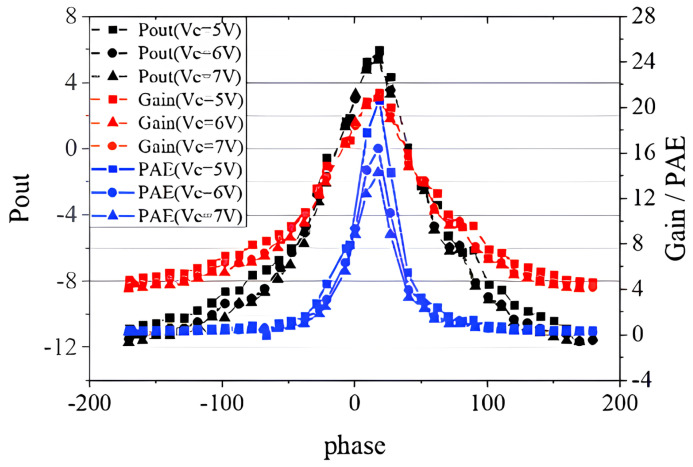
RF characteristics of the device at load mismatch.

To test the characteristics of the device under load mismatch, we first set up the VSWR circle with 50 Ohm as the center of the circle according to the conventional method. When VSWR = 10, the selected impedance points are shown in the red square in Figure 11. The output power and efficiency results are shown in Figure 12. It is obvious that the performance of the device was significantly reduced compared to the load at the optimum impedance since the output power was degraded to less than 6 dBm from 7.1 dBm. It can also be seen that the device achieved its best performance when the phase was around 20${}^{\circ }$. When $V_{C}=5$ V, the maximum output power was 5.95 dBm, which was 23.4% lower than when the load was matched; the maximum PAE was 20.67%, down about 29.3%; the maximum transducer gain was 21.03 dB, which was not much different from when the load was matched.

**Figure 13:**
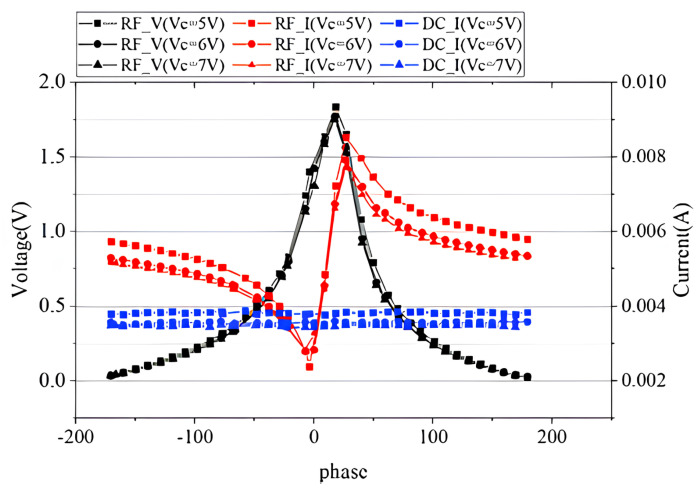
DC characteristics of the device at load mismatch.

In addition, we measured the dynamic RF voltage and current characteristic when the load was not matched. With the help of the newly introduced calibration method, the real-time RF voltage and current can be acquired to understand the breakdown mechanism of the transistors. The test results are shown in Figure 13. We can see that the RF current did not fluctuate greatly with the phase change, and the RF voltage and current fluctuated significantly with the phase change. When the phase was about 20${}^{\circ }$, the RF voltage reached its maximum value and dropped significantly with the increase or decrease of the phase. It was noticed that the collectorcurrent decreased slightly with the increase of $V_{C}$, which seemed to be caused by the annealing effect of the device. When $V_{C}$ was 8 V and the phase of the impedance was 20°, the device blew up, as shown in Figure 8b, which exactly verified that the maximum peak voltage at the collector could be the main cause of the HBT’s breakdown.

**Figure 14:**
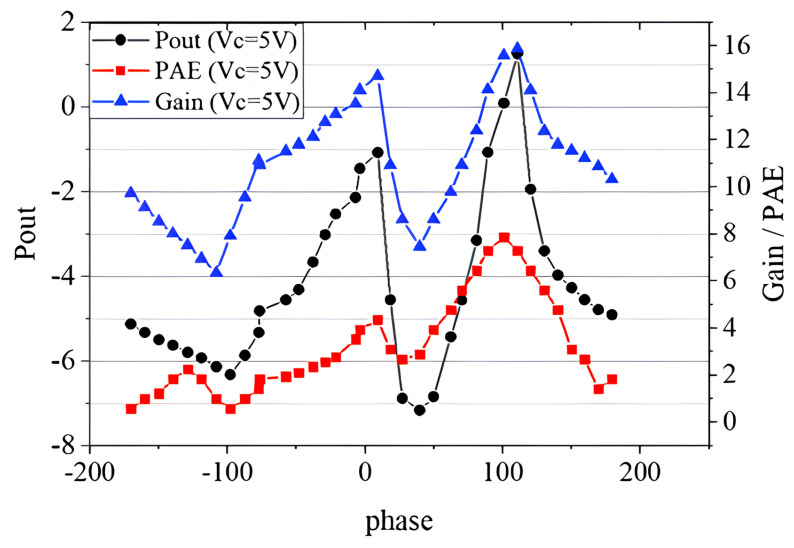
RF characteristics of the device at load mismatch.

**Figure 15:**
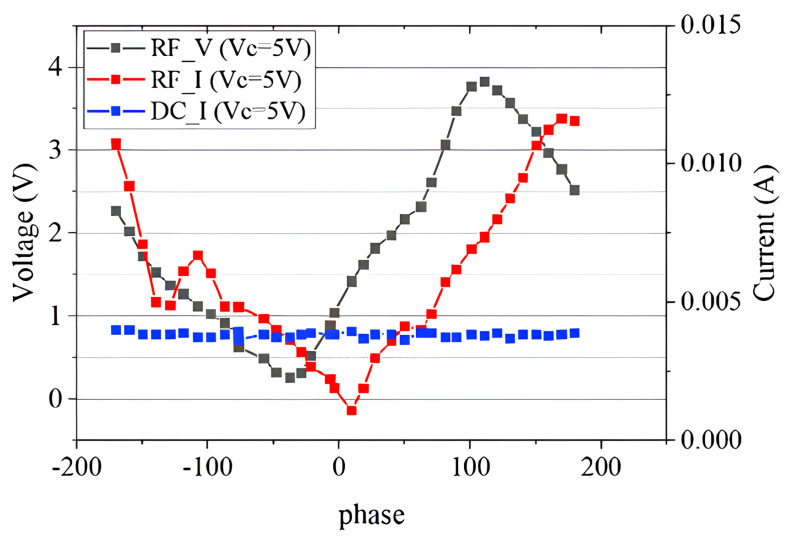
RF characteristics of the device at load mismatch.

In addition to the above conventional VSWR tests, we performed a load mismatch test using the VSWR circle centered on the optimal impedance point. The selected impedance points are shown as the blue dots in Figure 11. The test results are shown in Figure 14 and Figure 15, and the phase in the diagram is the phase in the Smith chart with the optimal impedance point as the center. It can be seen from Figure 14 that the device had the best performance when the phase was about 110${}^{\circ }$. The maximum output power was 1.25 dBm; the maximum PAE was 7.29%; the maximum transducer gain was 15.87 dB. Compared with the traditional load mismatch test results above, the optimal performance was greatly reduced. The breakdown occurred when the $V_{C}$ was 5.5 V and the phase was about 110${}^{\circ }$, which is consistent with the RF voltage reaching its maximum at 110${}^{\circ }$ in Figure 15. It can be seen that the voltage at which the device breakdown occurred in this case was also much lower than the voltage at which the breakdown occurred in the traditional load mismatch test. Figure 15 also shows that the maximum RF voltage and current were also significantly higher than the data from the traditional load mismatch test.

From the above comparison, it can be seen that the test results obtained by using a VSWR circle centered at 50 Ohm did not correctly reflect the impact of the load mismatch on the device in real applications, and the actual impact will be much worse. Therefore, in order to obtain accurate characteristics of the device in real applications, the load mismatch test should be performed around the optimal load impedance, which represents the load optimal impedance in real working conditions.

## 5. Conclusions

In this paper, we presented a novel method for measuring the VSWR’s ruggedness utilizing reverse wave injection technique with a real-time waveform measurement system. Through controlled power injection, we achieved rapid attainment of a synthesized load impedance approaching unity. The experimental validation illustrated that, under load mismatch conditions, the performance such as the RF output power, gain, and efficiency exhibited pronounced dependence on the load phase. Moreover, leveraging an almost unity sweep region enabled the extension of the non-50 Ohm-centered VSWR circle sweep test to the optimum impedance center, which is helpful to properly evaluate the robustness of a device around a complex impedance in real working conditions.

The introduction of reverse wave injection is helpful for understanding the device’s operational behavior in real-world scenarios. The analysis of the RF I–V curve further enabled a more-detailed localization of the region where the device was most susceptible to breakdown. This advancement not only aids in the progress of circuit technology, but also enhances the optimization of power amplifiers (PAs). Furthermore, these findings offer valuable insights for the design of non-isolated transceiver modules, thereby contributing significantly to the evolution of communication systems.

## Data Availability

Data sharing not applicable.
